# Ten Important Considerations for Ovarian Cancer Screening

**DOI:** 10.3390/diagnostics7020022

**Published:** 2017-04-13

**Authors:** Edward J. Pavlik

**Affiliations:** Division of Gynecologic Oncology, Department of Obstetrics and Gynecology, The University of Kentucky Chandler Medical Center and the Markey Cancer Center, Lexington, KY 40536-0293, USA; epaul1@uky.edu; Tel.: +1-859-323-3830; Fax: +1-859-323-1018

**Keywords:** ovarian, cancer, screening, considerations

## Abstract

The unique intricacies of ovarian cancer screening and perspectives of different screening methods are presented as ten considerations that are examined. Included in these considerations are: *(1) Deciding on the number of individuals to be screened; (2) Anticipating screening group reductions due to death; (3) Deciding on the duration and frequency of screening; (4) Deciding on an appropriate follow-up period after screening; (5) Deciding on time to surgery when malignancy is suspected; (6) Deciding on how screen-detected ovarian cancers are treated and by whom; (7) Deciding on how to treat the data of enrolled participants; (8) Deciding on the most appropriate way to assign disease-specific death; (9) Deciding how to avoid biases caused by enrollments that attract participants with late-stage disease who are either symptomatic or disposed by factors that are genetic, environmental or social; and (10) Deciding whether the screening tool or a screening process is being tested.* These considerations are presented in depth along with illustrations of how they impact the outcomes of ovarian cancer screening. The considerations presented provide alternative explanations of effects that have an important bearing on interpreting ovarian screening outcomes.

## 1. Introduction

Screening for different cancers, can appear similar; however, closer inspection reveals that there are considerable differences in approaches to cancer screening. This report focuses on the factors, issues and characteristics that uniquely distinguish ovarian cancer screening.

## 2. The Bare-Bones Basics of Screening

Cancer screening can be over-simplified so that it is conceived as the application of a test that discriminates malignancy. In general, the test for malignancy can be image-based or reagent-based. Image-based screening utilizes the identification of peculiar visual features not unlike correctly finding Waldo in an illustration that contains Waldo and other characters that may resemble Waldo to some degree [1]. Identification skills and sufficient time to complete the visual assignments are central to an image-based approach. Biomarker-based screening utilizes a chemical outcome which gives a result that discriminates malignancy, usually through a cut-off value above which malignancy becomes more likely. This approach can be thought of as asking the test for a “yes” vs. “no” answer about malignancy. This is best illustrated by the cut-off value of CA-125 (*cancer antigen 125*) for recurrent malignancy. However, one should be mindful that CA-125 becomes elevated by a variety of benign conditions [2]. Overlap in the outcome values of both malignant and non-malignant tests on both sides of the cut-off can occur with biomarker-based screening tests.

The key concept described above is “discrimination of malignancy”. In simple terms this implies finding malignancy at a high rate, missing malignancy at a low rate, and testing non-malignancy as malignancy at a low rate. To do this, protocols that test screening discrimination must be designed to assess screening effectiveness.

## 3. Collecting Evidence to Examine Screening Effectiveness—Perspective Analysis for a Prospective Screening Trial

### 3.1. Consideration 1

#### Deciding on the Number of Individuals to Be Screened

After the screening tool has been selected, the first step is to make decisions about the size of the screening group framed against a time period needed to accumulate that number of screens. This time frame must be long enough to include a sufficient number of incident cases to give the incident portion of the study power because it is the screening detection of incident cases that can be expected to be at an early stage and demonstrate the clearest benefit from screening. In the Kentucky Ovarian Screening trial, approximately half the malignancies detected by screening were incident [3], and this suggests that the sample size predicted apriori by power analysis probably should be twice as large as a power prediction based on both prevalent and incident cases. For simplicity, incident cases can be defined as those detections that occur after receiving at least one normal screen. This sample enlarged for incidence should be able to distinguish screening effectiveness in prevalent vs. incident cases. A key issue is utilization of a standard of significance to determine power and test results in order to guarantee reproducibility. By comparing Bayesian hypothesis testing with classical hypothesis tests, it has been reported that thresholds for a significance finding should be changed to *p* < 0.005 [4], however, doing such would increase sample size, duration of the trial and ultimately costs [5]. Others favor less stringency and including assessments of actual costs, benefits and probabilities [6]. A potential solution is possible by balancing a weighted sum of type I (false positive) and type II (false negative) errors [7,8]. Bearing in mind that the present status of the UK Collaborative Trial of Ovarian Cancer Screening (UKCTOCS) [9] is an inability to detect a significant statistical difference in survival between screened and unscreened women, the chance of not detecting a difference between groups must be respected [10] by the doubling of sample size as outlined above. Although other factors have been enumerated that are responsible for research findings that are false [11], they do not mitigate the mistake of insufficient power based on choosing too low a level of significance.

### 3.2. Consideration 2

#### Anticipating Screening Group Reductions due to Death

**Figure 1:**
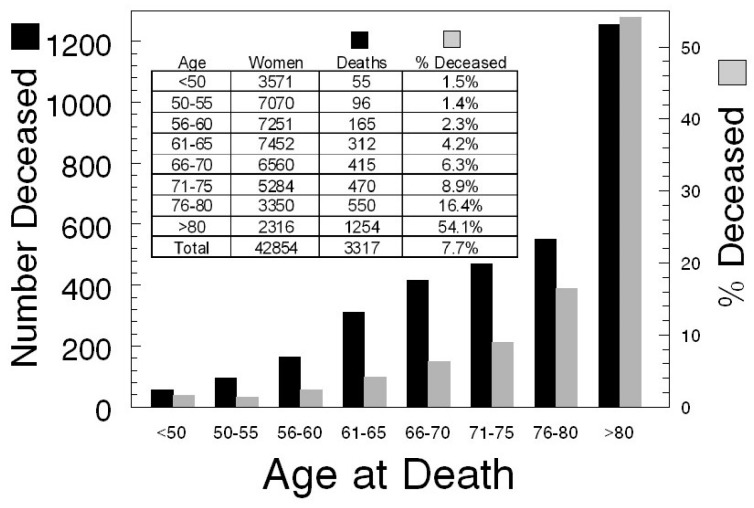
Age at death of screening participants.

Based on family reports and the Social Security Death Index (SSDI), 7.7% of 42,000+ participants in the Kentucky Ovarian Cancer Screening trial died after they began participating, with women over age 75 accounting for 70% of these deaths (Figure 1). Because of participants providing incorrect identifying information and due to the 3-year lag in listing on the SSDI, it is reasonable to expect an overall reduction in the screened population due to death of ~10%. Importantly, as the follow-up window extends to older age groups, a reduction in the screened population due to death of participants that can be followed for disease-specific survival will occur. This increase should be anticipated and used to adjust the group size predicted by power analysis.

### 3.3. Consideration 3

#### Deciding on the Duration and Frequency of Screening

The four major ovarian screening trials [3,9,12,13] used a periodic annual screening approach that accrued participants for 4.6–28.1 years and continued screening after enrollment for 7.7–28.1 years [14]. Two trials have employed a serial evaluation of abnormal screens [9,15]. Duration of the screening portion of the trial is a function of the sample needed and the resources made available to screen. A more difficult question regards frequency of screening. Screening high-risk women every six months has been practiced in the Kentucky trial without prior demonstration of benefit. The repeat screening interval after an abnormal screening exam is more subjective and has been performed at 3–6-month intervals on ovarian abnormalities that appear to be of low risk (cysts and cysts with septations) and at 4-week intervals for 3 months on ovarian tumors of uncertain malignant potential [16]. Annual follow-up for five years has been recommended for ovarian abnormalities that remain stable on several surveillance intervals of < 6 months [16].

A simplified picture of screening frequency is that women with a normal result be scheduled for annual screening, women with a result that is low risk for malignancy are screened more frequently and those with high risk for malignancy or with an abnormality of uncertain malignant potential are screened even more frequently. However, by what method can a result be assigned to one of these categories that minimizes subjectivity? Several characteristics are associated with an expected low risk grouping: (unilocular or septate morphology, morphology index (MI) = 4 or less, ΔMI less than 1.0/month, low-risk Assessment of Different NEoplasias in the adneXa or ADNEX score, absence of Doppler flow, CA125 (Cancer Antigen 125 <200 units/mL premenopausal or < 35 postmenopausal), CA125 stable/month, OVA1 (<5.0 premenopausal or <4.4 postmenopausal, OVA1 is the first multivariate index assay with FDA clearance), low-risk Risk of MAlignancy (ROMA) test, [17], absence of pelvic fluid), while others are associated with considering a high risk grouping (complex or solid morphology, MI >4, ΔMI (1.0/month or greater), high-risk ADNEX score, central Doppler flow, CA125 (≥200 units/mL premenopausal or ≥35 postmenopausal), CA125 (doubling within a month), OVA1 (≥5 premenopausal or ≥4.4 postmenopausal), high risk ROMA [17], pelvic ascites >60 cm^3^). These characteristics have been discussed with more definition in the context of low- and high-risk groups elsewhere [16]. When a new screening modality is decided upon, one or more of these characteristics should be employed for deciding the frequency of its application based upon a potential for risk of malignancy.

An abnormality of uncertain malignant potential may be considered as a tumor of indeterminate status. Following these abnormalities for either resolution or worsening status presents a logical rationale. The Kentucky Ovarian screening Program has activated a protocol to decide if continuing surveillance or a decision-favoring surgery will be made based on findings [18]. In this protocol, four risk groups are defined. Risk Group A (MI_0_ 0–2) is considered for surgery if the MI increases by 2 or more in the first 4 weeks of observation or 3 or more in the next 12 months. Risk Group B (MI_0_ 3–4) is considered for surgery if the MI increases by 1 or more in the first 4 weeks of observation or 2 or more in the next 12 months (observation at 3 & 12 months). Risk Group C (MI_0_ 5–6) is considered for surgery if the MI increases by 1 or more in the first 4 weeks of observation or 1 or more in the next 12 months (observation at 3, 6, 12 months). Risk Group D (MI_0_ 7–10) is considered for surgery if the MI increases by 1 or more or remains unchanged in the first 4 weeks of observation. Thus, this protocol utilizes variable periods of observation that are determined by the level of risk determined initially (MI_0_).

### 3.4. Consideration 4

#### Deciding on an Appropriate Follow-Up Period after Screening

An overly simple view of follow-up after screening is that it should extend long enough after the last participant in the screening trial has been screened to adequately assess the effect of screening on survival. However, a lesson learned from the UKCTOCS trial is that incident cancers occur after the first screen so that the follow-up for survival can be expected to be extended by one or more years. In the UKCTOCS trial, 4.6 years of screening accrual was coupled to 6.1 years of periodic screening and a final 3.1 years of follow-up. Secondly, over the course of a trial that occupies a decade of time, new treatments can be expected to be introduced that extend survival. Taken together, a longer follow-up extended to 10 years might be more appropriate for the UKCTOCS screening model to adequately assess the effect of screening on survival.

### 3.5. Consideration 5

#### Deciding on Time to Surgery When Malignancy Is Suspected

**Figure 2:**
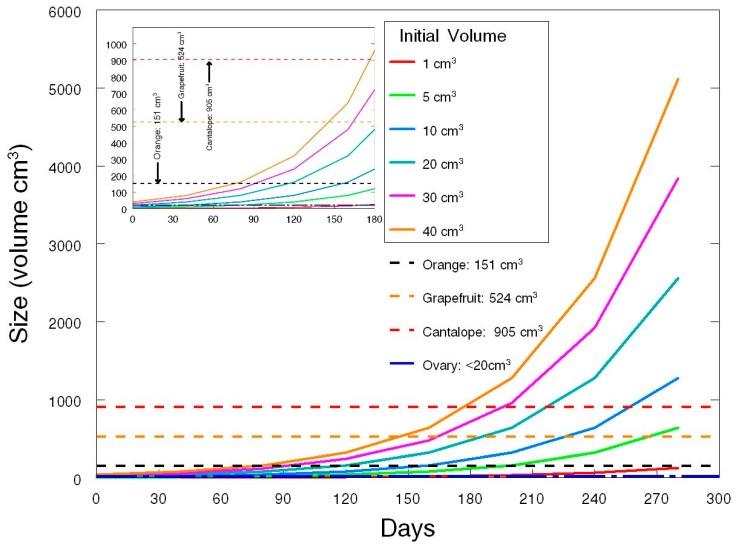
Ovarian Malignancy Doubling.

A 40-day tumor doubling time for ovarian malignancy has been estimated using the doubling of CA-125 [19]. While tumor doubling time may vary in different tumors, a 40-day doubling estimate is a good mid-range value [20]. Using this doubling time (Figure 2), comparative increases in size indicate that if the interval between a screen-detected abnormality and surgery is prolonged, tumor size will advance considerably. The mean volume of early stage ovarian malignancies (Stage I & II) detected by the Kentucky Ovarian Cancer Screening Program is 115 cm^3^ (±26.7 (SEM)). This represents enlargement to about 75% the size of an orange (Figure 2 black dashed line) and upon removal is associated with significantly extended survival. After 90 days, malignant tumors with an initial volume of up to twice the size of the ovary will approach or exceed the size of an orange and this indicates that the time to surgery should be limited to well under 90 days after a screening is decided to be indicative of malignancy. Efforts in the Kentucky Ovarian Cancer Screening Program limit the time to surgery to less than 30 days to minimize the opportunity for an early stage screening detection to develop into advanced disease diagnosed at surgery. In contrast, the Prostate, Lung, Colorectal and Ovarian Cancer (PLCO) Screening Trial [13] allowed the time to surgery to extend for up to 9 months, a duration that would allow very considerable increases in tumor burden and the opportunity for the development of disease diagnosed at an advanced stage.

### 3.6. Consideration 6

#### Deciding on How Screen-Detected Ovarian Cancers Are Treated and by Whom

It has recently been recognized that better outcomes are achieved when ovarian cancer is treated by specialists at high volume hospitals [21,22,23,24,25,26,27,28,29]. No provision for treatment by specialists in high volume hospitals was included in the PLCO trial [13]. Consequently, it is likely that the treatment component of this trial under-performed the detection component and accounted for less than optimal survivals. In order to reduce confounding factors due to treatment that could be deleterious for survival, an ovarian screening trial should limit treatment to high-volume centers by a gynecologic oncologist adhering to National Comprehensive Cancer Network guidelines so that optimal therapy based on staging will be provided. Doing so may be particularly appropriate for early stage ovarian cancer in order that chemotherapy can be utilized in high grade tumors [29,30].

### 3.7. Consideration 7

#### Deciding on How to Treat the Data of Enrolled Participants

In the PLCO trial [13], the UKCTOCS trial [9,31] and the Shizuoka Cohort Study of Ovarian Cancer Screening (SCSOCS) trial [12], enrollment in the screening arm was subject to intention to treat (ITT) analysis so that participants were analyzed in the group to which they were originally randomized: “once randomized an individual was always analyzed”, even if they were assigned to the screening arm, but never were screened or never received treatment. In this model anything that occurs after randomization is ignored, including non-compliance, protocol deviations, and withdrawal [32]. In contrast, in the Kentucky Ovarian Screening Program [3], only participants that completed the screening and treatment phases of the protocol were analyzed as a per protocol population. ITT analysis strongly favors preserving sample size so that originating power estimates continue to apply. The null hypothesis in a screening trial is that screening does not work. In the simplest sense, this null hypothesis is true if screening is falsely claimed to have a positive effect on disease, but positive screens cannot have a positive effect on disease if treatment is absent or sub-optimal. Individuals in the screening arm that do not receive screening and treatment will make the screening arm less distinguishable from the control arm, while individuals in the non-screening control arm that do receive screening and treatment will make the control arm less distinguishable from the screening arm. ITT analysis gives equal weight to each of these alternatives without testing for balance. Individuals who will seek out, schedule, attend and pay for screening are likely to occur less frequently than those who are assigned to the screening group but become non-compliant for receiving screens and treatment. This imbalance of never-screened individuals in the screening group is more likely to be greater than individuals who cross over to screening in the control group and will dilute the effectiveness of screening. This imbalance will not occur in a protocol-driven trial where unscreened/untreated individuals in the screening arm are censored, as well as individuals, screened independent of the protocol, in the unscreened control arm. In the PLCO trial, this imbalance consisted of 24 never-screened cases within the screening group, 21 untreated screen-positive cases in the screening group, and 8 cases in the screening group that were sub-optimally treated because they did not receive chemotherapy and accounted for 25% of the 212 malignancies reported in the screening group [13]. For the unscreened control arm, 25 untreated cases and 5 sub-optimally treated cases were reported or 17% of the 176 malignancies reported in the control arm [13]. No information was reported on how many cases in the control arm obtained treatment based on seeking access to the screening method. In summary, to test the question “Does screening work?” only cases of positive screens in the intervention group should be included that received treatment adhering to National Comprehensive Cancer Network guidelines, while the control group should identify and censor cross-over cases that obtained out-of-protocol screening.

The PLCO investigators decided to interpret the interval of protection conferred by screening to extend considerably beyond one year. Re-examination of the PLCO data by other investigators that limited the analysis to cancers detected within one year of screening showed that the survival in the screening group was significantly better than in the control group (*p* = 0.0017) and contained fewer Stage IV cases [33]. Thus, it is important to realize that malignancies that appear several years after screening should not be included in the intervention group, and should be censored as an “out-of-screening cycle” event.

### 3.8. Consideration 8

#### Deciding on the Most Appropriate Way to Assign Disease-Specific Death

Facile assignment of mortality due to disease is death that occurs with evidence of disease while under treatment for ovarian cancer, meeting the requirement used in both the PLCO and UKCTOS trials that *the disease process and/or associated treatments initiated or sustained a chain of events causally responsible for death*. Conversely, a sudden death with no evidence of disease is a death clearly due to other causes. Conditions for assigning disease-specific death are complicated when disease is evident and a sudden death occurs. Accidents, suicide, diabetic death, stroke and cardiac failure may be responsible for these complications. Difficult assignments of cause of death occur when reporting is incomplete. Both the PLCO trial and the UKCTOCS trial adjudicate disease-specific death differently [34,35]. The PLCO trial incorporated efforts to determine the underlying cause of death through periodic updates of questionnaires, cancer registries, and attempted contacts with next-of-kin and personal physicians (Table 1). Different procedures were used after the first two years of the PLCO trial to ascertain the underlying cause of death. The global resource available to the PLCO trial was the National Death Index which restricts the release of information until three years after any death has occurred. Admittedly, more information was available to the PLCO trial for screened cancers than for unscreened cancers and the control group [35]. The UKCTOCS trial had much greater global access to cancer and death registrations using the National Health Service (NHS) number of participants to access information from the Health & Social Care Information Center, the National Cancer Intelligence Network, Hospital Episodes Statistics, Central Services Agency, the Northern Ireland Cancer Registry, and the Hospital Episodes Statistical records (Table 2). To resolve the underlying cause of death, two pathologists and two gynecologic oncologists relied upon an algorithm involving disease progression (new lesions or increase in size of original lesions by imaging), clinical worsening, or rising biomarkers. Clearly the UKCTOCS had a more comprehensive access to death and factors related to cause of death through information arising in national health services.

### 3.9. Consideration 9

#### Deciding How to Avoid Biases Caused by Enrollments that Attract Participants with Late-Stage Disease Who Are either Symptomatic or Disposed by Factors that Are Genetic, Environmental or Social

It may be possible to explain the failure to detect early stage disease in the PLCO trial in terms of promotions that attracted symptomatic women or women already with late-stage disease. If recruitment inadvertently allowed a biased enrollment of women who already were demonstrating clinical disease, it would certainly explain why early stage disease was not detected. Such a bias could also be contributed to by attracting nulliparous women or women with a family history of ovarian cancer. In contrast to the PLCO trial, the UKCTOCS ran a separate protocol specialized for women at elevated risk for ovarian cancer. Since screening is intended for detecting sub-clinical disease, post-hoc analysis should be performed that censors participants with clinical manifestations of disease when the screening tool is not needed.

### 3.10. Consideration 10

#### Deciding Whether the Screening Tool or a Screening Process Is Being Tested

Differences in the screening process between the PLCO and UKCTOCS trials have already been outlined here and in print [14,36] and are likely to have greater impact on outcomes than differences in the screening tools in these two trials. As an aside, completion of full human papillomavirus (HPV) vaccination is subject to age, rural vs. urban location, parental hesitancy/refusal and cultural factors [37,38]. In this example, which utilizes a very effective agent, effectiveness at the population level is limited by these barriers to utilization so that the role of the process assumes great importance even with a very effective vaccination tool. In summary, in a screening trial both the screening tool and the screening process contribute to the overall evaluation so that it is possible for a quite effective screening tool to be utilized in a flawed screening process with the result that overall outcomes are unimpressive. As part of this consideration, the control group is also process driven. If the control group is supposed to receive “usual care”, such care could involve no visits to a care-giver as well as timed annual visits that are matched to the frequency of screening visits. In this latter case, the scheduled visits may provide a superior level of care that, based on information related by the subject, leads to imaging with CT or MRI and the potential to identify malignancy. Against this background it is not surprising that individuals in the control arm of clinical trials do better than the overall population.

## 4. Conclusions

Ten considerations are presented here that can impact the outcomes of ovarian cancer screening. Each should be considered for implementing screening processes and re-considered in post-hoc analyses as alternative explanations of effects that influence screening outcomes.

In addition, the consideration of ovarian cancer risk is appropriate and has been coupled to ovarian cancer screening. The United Kingdom Familial Ovarian Cancer Screening Study (UKFOCS) was begun in 2007 and included 4348 women that received annual screening for five years and follow-up for an additional 4.8 years [39]. The participants met the familial criteria for risk by having had a family member that had been diagnosed with ovarian cancer and would be considered to have a life-time risk ≥10%. A shift to early-stage ovarian cancer discovery was observed to result from this screening; however, it is too early to tell if an improved survival will be demonstrated in this screened group of high-risk women. Improved assessments of risk have now been defined based on mutations in *BRCA*_1_ (Breast Cancer susceptibility gene 1: 39%–65% life-time risk), and *BRCA*_2_ (Breast Cancer susceptibility gene 2: 11%–37% life-time risk) [40,41]. Additional germline mutations in *BRIP*_1_, *BARD*_1_, *PALB*_2_, *NBN*, *RAD51B*, *RAD51C*, *RAD51D* [42,43] as well as *MLH*_1_, *MSH*_2_, *MSH*_6_, *PMS*_2_, *EPCAM*, (all associated with Lynch syndrome [44]), *TP*_53_ (associated with Li-Fraumeni syndrome [45]) and *STK*_11_/*LKB*_1_ (associated with Peutz-Jeghers syndrome [46]) are related to moderately increased risk of ovarian cancer. With the number of germline mutations expanding, there has been support for population-based screening for all women before ovarian cancer develops [47]. Such a position would allow surveillance screening, surgical prophylaxis, or chemoprevention through oral contraceptives. However, utilization of these strategies must be weighed against potential problems (false negative screening, surgical complications, stroke, pre-mature menopause and increasing the risk of other cancers). Thus, with the list of associated gene mutations evolving, more women can be expected to carry some mutation pre-disposing them to ovarian cancer and overall will exceed the 15% of all ovarian cancers attributed to *BRCA*_1_ and *BRCA*_2_ [46]. In this context, some form of ovarian cancer screening/surveillance will have a role.

## Figures and Tables

**Table 1:** Mortality review in the Prostate, Lung, Colorectal and Ovarian Cancer Screening (PLCO) trial.

PLCO	PLCO	PLCO
Death due to ovarian cancer	The disease process and/or associated treatments initiated or sustained a chain of events causally responsible for death	Identify other underlying cause of death
Annual update questionnaire	Periodic	
Population-based cancer registries	Whenever possible	
Linkage to National Death Index	Periodic	
Obtained diagnostic medical records: Abstracted by registrars: stage, histology , grade, and treatment	Reviewers blinded to participation in screened vs. unscreened arm	Identify next of kin and personal physician
Underlying cause of death: first 2 years	Death certificate & relevant determinations underlying cause of death	Potential, ovarian cancer deaths, deaths of unknown or uncertain deaths were reviewed by at least 1 member of a panel of expertise (2 reviewers with discrepancies decided by a third)
Underlying cause of death: after year 2	Primary reviewer considered records without access to death certificate	If primary review disagreed with death certificate, a second expert reviewed record & death certificate. Disagreement triggered another independent review which led to a resolution by meeting or teleconference
Attempt to collect identical death information from both screen-detected and non-screen detected cancers	Screen-detected cancers will have more extensive information collected	Less information for both unscreened group participants & screened false positives

**Table 2:** Mortality review in the UK Collaborative Trial of Ovarian Cancer Screening (UKCTOCS) trial.

UKCTOCS	UKCTOCS	UKCTOCS
Direct communication with participants		
Postal follow-up questionnaires	3–5 years after randomization	
Diagnosis: England & Wales	Linked by NHS number to the Health & Social Care Information Center, the National Cancer Intelligence Network, Hospital Episodes Statistics	Cancer & death registrations
Diagnosis: Northern Ireland	Central Services Agency and the Northern Ireland Cancer Registry	Cancer & death registrations
Surgery outside the trial	Hospital Episodes Statistical records	
Underlying cause of death	Outcomes review committee (2 pathologists & 2 gynecological oncologists)	Final diagnosis based on algorithm: disease progression, (new lesions or increase in size of original lesions by imaging, clinical worsening, or rising biomarkers)
